# Multi-User Measurement-Device-Independent Quantum Key Distribution Based on GHZ Entangled State

**DOI:** 10.3390/e24060841

**Published:** 2022-06-18

**Authors:** Ximing Hua, Min Hu, Banghong Guo

**Affiliations:** 1 Guangdong Provincial Key Laboratory of Nanophotonic Functional Materials and Devices, Guangdong Provincial Key Laboratory of Quantum Engineering and Quantum Materials, South China Normal University, Guangzhou 510006, China; 2019022062@m.scnu.edu.cn; 2 National Quantum Communication (Guangdong) Co., Ltd., Guangzhou 510535, China; 3 Key Laboratory of Quantum Information, University of Science and Technology of China, Chinese Academy of Sciences, Hefei 230026, China

**Keywords:** quantum key distribution, GHZ entangled state, measurement-device-independent, multi-user

## Abstract

As a multi-particle entangled state, the Greenberger–Horne–Zeilinger (GHZ) state plays an important role in quantum theory and applications. In this study, we propose a flexible multi-user measurement-device-independent quantum key distribution (MDI-QKD) scheme based on a GHZ entangled state. Our scheme can distribute quantum keys among multiple users while being resistant to detection attacks. Our simulation results show that the secure distance between each user and the measurement device can reach more than 280 km while reducing the complexity of the quantum network. Additionally, we propose a method to expand our scheme to a multi-node with multi-user network, which can further enhance the communication distance between the users at different nodes.

## 1. Introduction

Quantum key distribution (QKD) allows two users, Alice and Bob, to share a secure key privately [1,2]. The first QKD protocol, called the BB84 protocol, was proposed in 1984 by Bennett and Brassard [3]. However, because of the gaps between reality and theory, there exist various loopholes in practical systems through which eavesdroppers can attack the QKD process [4]. Therefore, several investigators have focused on finding ways to resist such attacks [5,6]. In 2012, Lo et al. proposed a measurement-device-independent quantum key distribution (MDI-QKD) protocol [7] to prevent attacks on measurement devices and enhance the communication distance between two users.

QKD research usually begins with a point-to-point scheme. With the development of quantum networks [8,9,10], multi-user scenarios have become research hotspots. Multi-user QKD, known as quantum cryptography conference (QCC) such as Greenberger–Horne–Zeilinger (GHZ) states [11] based scheme [12] and measurement-device independent scheme [13] or quantum conference key agreement (CKA) such as the intensity-encoded scheme [14] and the scheme based on a W-class state [15], is effective in scenarios where multiple users share common secure keys. Multi-particle entangled states can easily apply in multi-user QKD realization [16], although the communication distance is limited by the stability of entangled states and other issues lead to such schemes being inferior compared to the existing single-photon interference schemes [17].

Ref. [18] proposed an MDI-QKD scheme with an entangled source in the middle and realized ultra-long communication. Inspired by the scheme, we propose a multi-user MDI-QKD scheme based on the GHZ entangled state. We analyze the security of our scheme and derive the secure key rate when users employ an ideal single photon source and a weak coherent source. The simulation results show that a multi-user MDI-QKD system can be realized under this scheme with a reduced number of detectors and quantum channels compared with traditional MDI-QKD, while the distance between each user and the measurement device can reach more than 280 km (more than 560 km between each two users). Additionally, we propose a method to expand our scheme to a multi-node with multi-user network, which can further enhance the communication distance between the users at different nodes. This paper is organized as follows: in Section 2, we introduce the multi-user MDI-QKD based on GHZ entangled state protocol. In Section 3, we estimate the performance of our scheme for an ideal single-photon source and a weak coherent source. In Section 4, we introduce a method to expand our scheme to a multi-node with multi-user network. Finally, a summary is presented in Section 5.

## 2. Protocol

Before providing details of our protocol, we simply introduce the background knowledge of our scheme. A GHZ entangled state is multi-particle entangled state in which each particle is entangled with other particles. It has maximum output mutual information, and resistance to white noise. The n-particle GHZ entangled state can be expressed as follows [19]:

(1) $$|\phi^{+}\rangle =\frac{1}{\sqrt{2}}{\left(|000\ldots 00\rangle +|111\ldots 11\rangle \right)}_{N}$$

Based on the distribution of an n-particle GHZ entangled state [16], n users can obtain the common secure key simultaneously. This leads to the generation of a secure key according to the measurement result of the GHZ entangled state.

In MDI-QKD [7], the measurement device utilizes the Hong–Ou–Mandel (HOM) [20] effect to construct the relationship between two input particles. According to the click of the detectors, we obtain the BSM result $|\psi^{+}\rangle =\left(|H\rangle |V\rangle +|V\rangle |H\rangle \right)/\sqrt{2}$ and $|\psi^{-}\rangle =\left(|H\rangle |V\rangle -|V\rangle |H\rangle \right)/\sqrt{2}$. In the Z basis, the successful BSM event ($|\psi^{+}\rangle$ and $|\psi^{-}\rangle$) represents the polarization of two particles being different, while the X basis $|\psi^{+}\rangle$ ($|\psi^{-}\rangle$) represents the two particles having the same (different) polarization.

Combining the distribution of the GHZ entangled state with the MDI-QKD, we can realize the multi-user QKD by entanglement swapping [21].

**Figure 1:**
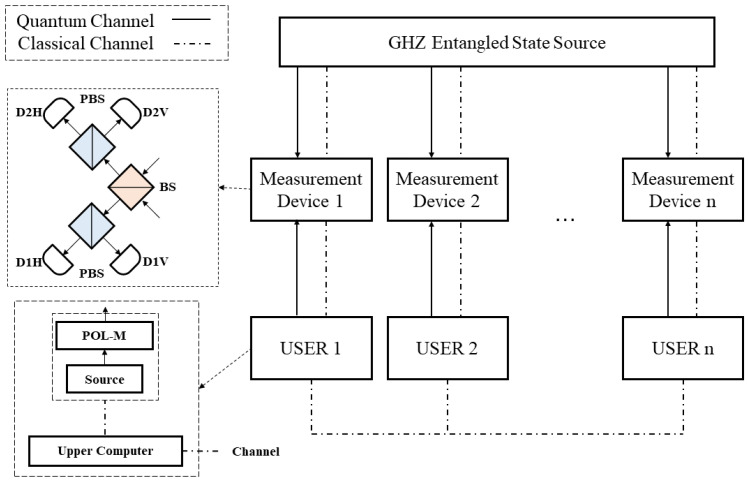
Schematic diagram of n-user MDI-QKD system. **POL-M**: polarization modulator; **BS**: beam splitter; **PBS**: polarization beam splitter; **D1H**, **D1V**, **D2H**, **D2V**: single-photon detector; **Source**: single-photon source or weak coherent source. We use solid line to depict the quantum channel and dotted line to depict the classical channel. The measurement device includes a BS, two PBSs, and four single-photon detectors, and implements the Bell state measurement (BSM) the same as the polarization-based MDI-QKD protocol. The GHZ-ESS can prepare GHZ entangled states with different numbers of particles corresponding to the number of users.

As depicted in Figure 1, there is a case of an n-user MDI-QKD system based on GHZ entangled states. The system can divide into four parts: user, measurement device, GHZ entangled state source (GHZ-ESS) and channel. The n-user system includes n users, n measurement devices, a GHZ-ESS, and channel. The user mainly contains a source, a polarization modulator, and a upper computer. In practice, we usually use a weak coherent source with a decoy-state instead of a single photon source. The user uses a polarization modulator to modulate BB84 polarization states. The upper computer control the source, the polarization modulator and the information processing. The information processing includes sifting the efficient data, post-processing, etc. Each user has a corresponding measurement device. The GHZ-ESS connects to all measurement devices. The number of particles in the GHZ entangled state is similar to the number of users. Each particle of the GHZ entangled state will interfere with the polarization state prepared by users in the corresponding measurement device. They will perform Bell state measurement (BSM) in the measurement device. The channel including quantum channel and classical channel. Quantum channels are used to transmit quantum signals. Classical information such as the basis of prepared polarization state is transmitted in classical channel.

**Figure 2:**
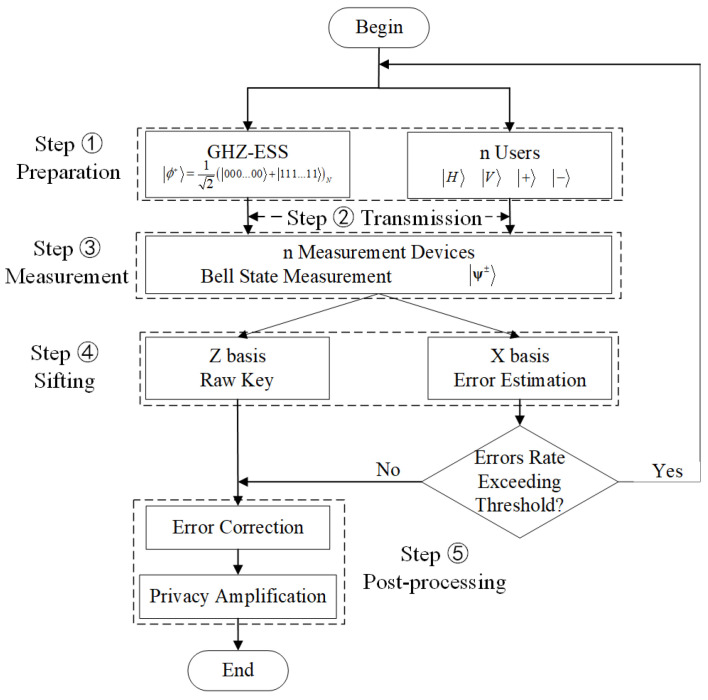
Flow chart of our protocol.

As shown in Figure 2, our protocol consists of five main steps:

**Step 1:** Preparation. Each user randomly prepares one of the BB84 states such as $|+\rangle$,$|-\rangle$ in the X basis or $|H\rangle$,$|V\rangle$ in the Z basis, while the GHZ-ESS randomly prepares an n-particle GHZ entangled state. The number of particles in the GHZ entangled state is equal to the number of users (in principle, each user only sends one photon).

**Step 2:** Transmission. Users (the GHZ-ESS) transmit the BB84 state (the GHZ entangled state) in different quantum channels between each user (the GHZ-ESS) and measurement device.

**Step 3:** Measurement. The measurement device performs BSM on the BB84 state and the GHZ entangled-state particles. Each particle of the GHZ entangled quantum state can interfere with a particle sent by its corresponding user. If there are only two detectors responding we call it a successful BSM event (a click in D1H and D2V, or in D1V and D2H are $|\psi^{-}\rangle$; a click in D1H and D1V, or in D2H and D2V are $|\psi^{+}\rangle$), similar to traditional MDI-QKD.

**Step 4:** Sifting. All users retain the bits when all the corresponding measurement device generates a successful BSM event. All users announce the basis of the prepared BB84 state, and the GHZ entangled source broadcasts the GHZ state it has prepared through the classical channels. In our scheme, only the states in the Z basis are used to generate a secure key; the states in the X basis are used to estimate the error rate. Hence, users retain the data prepared in the same basis by all users and discard the remaining data. Then, each user should either flip or not flip its local bits according to the BSM result, the GHZ states, and the prepared basis; see following and Appendix A for details. At this point, each user obtains the raw key.

**Step 5:** Post-processing. Similar to traditional point-to-point QKD protocols, users perform post-processing under the control of upper computer, which includes error correction and privacy amplification. They finally obtain the same secure keys.

Following the original MDI-QKD protocol [7], users need to operate on their local bits based on the GHZ entangled state and the BSM results to generate secure keys. Taking three users (named Alice, Bob, and Charles) as an example, Table 1 shows the relationship between the prepared GHZ state, the BSM results, and the operations of the three participants. The users can retain the signal to generate a secure key only when all the BSM results are $|\psi^{+}\rangle$ or $|\psi^{-}\rangle$.

For example, when the state prepared by the GHZ-ESS is $\frac{1}{\sqrt{2}}\left(|H\rangle |H\rangle |H\rangle \pm |V\rangle |V\rangle |V\rangle \right)$, the message can be retained as a sifted key only if Alice, Bob, and Charles have prepared the same polarization state and all the BSM results are $|\psi^{+}\rangle$ or $|\psi^{-}\rangle$.

## 3. Secure Key Rate

Next, we derive the secure key and error rates to investigate the performance of our scheme with a single-photon source and a weak coherent source.

By combining the MDI-QKD technique [7] and the GLLP method [22], the security key rate is given by

(2) $$R=Q_{1}^{Z}\left[1-H\left(e_{1}^{X}\right)\right]-Q^{Z}fH\left(E^{Z*}\right)$$

where $Q^{Z}$ and $E^{Z*}$ denote the gain and quantum bit error rate (QBER) in the Z basis, respectively; *f* is the inefficiency function for the error correction process; $Q_{1}^{Z}$ denotes the gain when all users send a single-photon state; $e_{1}^{X}$ denotes the phase error rate; and $H\left(x\right)=-x\log_{2}\left(x\right)-\left(1-x\right)\log_{2}\left(1-x\right)$ is the binary Shannon entropy function. $E^{Z*}=$ max $\left\{E_{U_{1}U_{2}}^{Z},E_{U_{1}U_{3}}^{Z}\ldots E_{U_{1}U_{n}}^{Z}\right\}$, where $E_{U_{1}U_{2}}^{Z}\left(E_{U_{1}U_{3}}^{Z}\ldots E_{U_{1}U_{n}}^{Z}\right)$ is the marginal quantum bit error rate between user 1 and user 2 (3…n) in the Z basis. In practice, $Q^{Z}$ and $E^{Z*}$ can be obtained from experimental data.

### 3.1. Key Rate of Single-Photon Source

For simplicity, we consider the case of three users in our scheme and estimate the secure key rate for a single photon source.

When the users use an ideal single-photon source to prepare the BB84 state, the gain in the Z basis is

(3) $$Q^{Z}=Q_{1}^{Z}=Y_{1}$$

where $Y_{1}$ denotes the probability of obtaining a successful BSM when all the users send a single-photon state. The yield $Y_{1}$ is given by

(4) $$\begin{array}{cc} Y_{1} & =\frac{1}{64}\{{\left(1-P_{d}\right)}^{2}\left[1-\left(1-P_{d}\right)\left(1-\eta \right)\right]\left[1-\left(1-P_{d}\right)\left(1-\eta_{d}\right)\right]\\ & +2P_{d}{\left(1-P_{d}\right)}^{2}\left[1-\left(1-P_{d}\right)\left(1-\eta \right)\left(1-\eta_{d}\right)\right]{\}}^{3} \end{array}$$

where $P_{d}$ is the dark count, $\eta_{d}$ is the detection efficiency, $\eta =\eta_{l}\times \eta_{b}=10^{-\alpha L/10}\times \eta_{b}$ is the transmittance between the users and measurement device, and $\eta_{l}$ represents the channel loss. $\frac{1}{64}$ represents the possibility that users send similar or different polarization states to the particles of the GHZ entangled state in the Z basis.

We assume that our entangled source is perfect; therefore, the error rate contains two main contributions: (1) the error rate $e_{0}$ caused by background counts and (2) the error rate $e_{d}$ corresponding to the misalignment and instability of the optical system. The total error rate is as follows:

(5) $$E^{Z*}=E_{U_{1}U_{2}}^{Z}=E_{U_{1}U_{3}}^{Z}=e_{0}-\frac{\left(e_{0}-e_{d}\right)\left(\frac{1}{16}\eta^{2}\eta_{d}^{2}{\left(1-P_{d}\right)}^{4}\right)}{Y_{U_{1}U_{2}}}$$

where $Y_{U_{1}U_{2}}$ denotes the probability of both measurement devices obtaining a successful BSM when user 1 and user 2 send a single-photon state. Similarly, we obtain the error rate in the X basis as follows:

(6) $$e_{1}^{X}=e_{0}-\frac{\left(e_{0}-e_{d}\right)\left(\frac{1}{64}\eta^{3}\eta_{d}^{3}{\left(1-P_{d}\right)}^{6}\right)}{Y_{1}}$$

**Figure 3:**
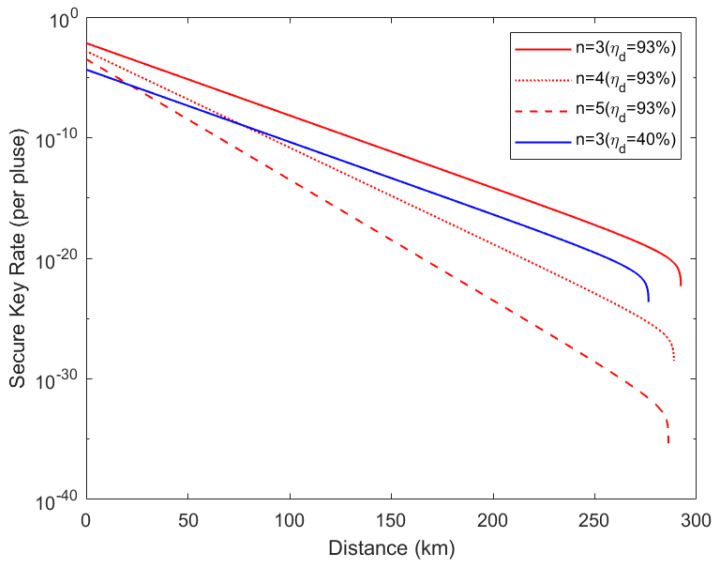
Secure key rate with single-photon state. The red line corresponds to the detection efficiency $\eta_{d}$ of 93% [24], and the blue line corresponds to the detection efficiency $\eta_{d}$ of 40% [23]. **n** is the number of communication users. The distance refers to the length of the quantum channel between any user and the measurement device.

Utilizing the experimental parameters in Table 2 [23], we obtained the simulation results shown in Figure 3. These results show that the communication distance between each user and the GHZ-ESS can exceed 280 km using optical fibers. Using an ideal single-photon source and a perfect GHZ-ESS, the GHZ-ESS can be located at the center to establish a star-configuration quantum network with a radius of 280 km.

### 3.2. Key Rate of Weak Coherent Source with Decoy State

In this section, we analyze the realization of the decoy state using our protocol. Users use the decoy state to resist a photon number splitting (PNS) attack on a weak coherent source. In our protocol, we use the Z basis to generate the secure key and the X basis to detect the error bit. Therefore, we take the quantum state in the Z basis as the signal state (only preparing the signal state) and that in the X basis as the decoy state (preparing both signal state and decoy state).

In our analysis, two decoy-state techniques (signal state *v*, vacuum state $\mu_{2}$, decoy-state $\mu_{1}$) are used, where $v>\mu_{1}>\mu_{2}=0$ represents the mean photons of the sources.

We consider the situation consisting of three users as an example to derive the secure key rate. According to the decoy state method [25], in the Z basis, for each measurement device, we can estimate the gain $Q_{m}^{Z}$ and error rate $E_{m}^{Z}$ as follows:

(7) $$Q_{m}^{Z}=\sum_{i=0}^{\infty }e^{-v}Y_{1i}\frac{v^{i}}{i!}$$

(8) $$E_{m}^{Z}Q_{m}^{Z}=\sum_{i=0}^{\infty }e^{-v}e_{1i}Y_{1i}\frac{v^{i}}{i!}$$

where $Y_{1i}$ ($e_{1i}$) represents the yield (error rate) from the GHZ-ESS and the corresponding user. The subscript 1 that different from the traditional MDI-QKD [7] represents the particle of GHZ entangled state in each measurement device. Therefore, we can estimate the total gain and error rate in the Z basis as follows:

(9) $$Q^{Z}=\sum_{i=0}^{\infty }\sum_{j=0}^{\infty }\sum_{k=0}^{\infty }e^{-3v}Y_{111ijk}\frac{v^{i+j+k}}{i!j!k!}$$

(10) $$E^{Z}Q^{Z}=\sum_{i=0}^{\infty }\sum_{j=0}^{\infty }\sum_{k=0}^{\infty }e^{-3v}e_{111ijk}Y_{111ijk}\frac{v^{i+j+k}}{i!j!k!}$$

where $Y_{111ijk}$ ($e_{111ijk}$) represents the overall yield (error rate) in the Z basis. We optimized the formula in the Ref. [26] and added the subscript 111 to describe the influence of GHZ entangled state. We Similarly, we can obtain the total gain and error rate in the X basis.

Because we use the decoy state technique in the X basis, we can obtain the total gain in the Z basis when all users send a single photon pulse as

(11) $$Q_{1}^{Z}=v^{3}e^{-3v}Y_{1}^{Z}$$

where $Y_{1}^{Z}$ represents the yield when all users send a single photon pulse.

Next, we need to estimate the lower bound of the yield and the upper bound of the error rate that each user sends for a single-photon pulse in the X basis. According to the decoy-state method [25,26,27], we can estimate that

(12) $$\begin{array}{cc} Y_{1}^{X}\geq & \frac{1}{v^{3}{\mu_{1}}^{3}\left(v-\mu_{1}\right)}[v^{4}(e^{3\mu_{1}}Q_{111\mu_{1}\mu_{1}\mu_{1}}^{X}-e^{2\mu_{1}}Q_{111\mu_{1}\mu_{1}0}^{X}-e^{2\mu_{1}}Q_{111\mu_{1}0\mu_{1}}^{X}-e^{2\mu_{1}}Q_{1110\mu_{1}\mu_{1}}^{X}\\ & +e^{\mu_{1}}Q_{111\mu_{1}00}^{X}+e^{\mu_{1}}Q_{1110\mu_{1}0}^{X}+e^{\mu_{1}}Q_{11100\mu_{1}}^{X}-Q_{111000}^{X})\\ & -{\mu_{1}}^{4}(e^{3v}Q_{111vvv}^{X}-e^{2v}Q_{111vv0}^{X}-e^{2v}Q_{111v0v}^{X}-e^{2v}Q_{1110vv}^{X}\\ & +e^{v}Q_{111v00}^{X}+e^{v}Q_{1110v0}^{X}+e^{v}Q_{11100v}^{X}-Q_{111000}^{X}) \end{array}$$

(13) $$\begin{array}{cc} e_{1}^{X}\leq & \frac{1}{{\mu_{1}}^{3}Y_{1}^{X}}(e^{3\mu_{1}}E_{111\mu_{1}\mu_{1}\mu_{1}}^{X}Q_{111\mu_{1}\mu_{1}\mu_{1}}^{X}-e^{2\mu_{1}}E_{111\mu_{1}\mu_{1}0}^{X}Q_{111\mu_{1}\mu_{1}0}^{X}-e^{2\mu_{1}}E_{111\mu_{1}0\mu_{1}}^{X}Q_{111\mu_{1}0\mu_{1}}^{X}\\ \; & -e^{2\mu_{1}}E_{1110\mu_{1}\mu_{1}}^{X}Q_{1110\mu_{1}\mu_{1}}^{X}+e^{\mu_{1}}E_{111\mu_{1}00}^{X}Q_{111\mu_{1}00}^{X}+e^{\mu_{1}}E_{1110\mu_{1}0}^{X}Q_{1110\mu_{1}0}^{X}\\ \; & +e^{\mu_{1}}E_{11100\mu_{1}}^{X}Q_{11100\mu_{1}}^{X}-E_{111000}^{X}Q_{111000}^{X}) \end{array}$$

where $Q_{111ijk}^{X}$ (*i*,*j*,*k* = 0, $\mu_{1}$, *v* represent the mean photon number intensities of users’ sources) is the overall gain in the X basis when users choose the corresponding intensities. $E_{111ijk}^{X}$ is the overall error rate in the X basis when users choose corresponding intensities.

**Figure 4:**
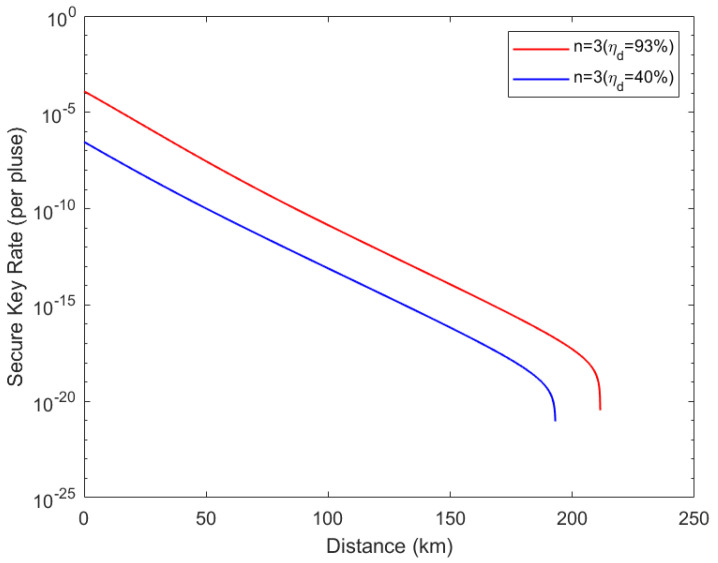
Secure key rate with weak coherent source. The simulation performed for the situation of three users had detection efficiency results of $\eta_{d}=93\%$ and $\eta_{d}=40\%$. The mean photons of signal state v=0.48, and the mean photons of decoy-state $\mu_{1}=0.05$. The distance refers to the length of the quantum channel between any user and measurement device.

Finally, utilizing the experimental parameters in Table 2, we can obtain the performance of our protocol when three users use weak coherent sources as shown in Figure 4. The simulation results show that the communication distance between each user and the GHZ-ESS can reach further than 210 km using optical fibers. Compared with a single photon source, the weak coherent source has a lower secure key rate and shorter communication distance while still realizing a signal transmission of more than 420 km between each two users.

### 3.3. Security and Discussion

In this section, we will analyze the security of our protocol and compare our protocol with other schemes.

Without loss of generality, we can depict the three-particle GHZ entangled state in eight orthogonal GHZ states as [26]:

(14) $$\begin{array}{c} |\alpha_{1}\rangle =\frac{1}{\sqrt{2}}\left(|H\rangle |H\rangle |H\rangle +|V\rangle |V\rangle |V\rangle \right)=\frac{1}{2}\left(|+++\rangle +|+--\rangle +|-+-\rangle +|--+\rangle \right)\\ |\alpha_{2}\rangle =\frac{1}{\sqrt{2}}\left(|H\rangle |H\rangle |H\rangle -|V\rangle |V\rangle |V\rangle \right)=\frac{1}{2}\left(|++-\rangle +|+-+\rangle +|-++\rangle +|---\rangle \right)\\ |\alpha_{3}\rangle =\frac{1}{\sqrt{2}}\left(|V\rangle |H\rangle |H\rangle +|H\rangle |V\rangle |V\rangle \right)=\frac{1}{2}\left(|+++\rangle +|+--\rangle -|-+-\rangle -|--+\rangle \right)\\ |\alpha_{4}\rangle =\frac{1}{\sqrt{2}}\left(|V\rangle |H\rangle |H\rangle -|H\rangle |V\rangle |V\rangle \right)=\frac{1}{2}\left(|++-\rangle +|+-+\rangle -|-++\rangle -|---\rangle \right)\\ |\alpha_{5}\rangle =\frac{1}{\sqrt{2}}\left(|H\rangle |V\rangle |H\rangle +|V\rangle |H\rangle |V\rangle \right)=\frac{1}{2}\left(|+++\rangle -|+--\rangle +|-+-\rangle -|--+\rangle \right)\\ |\alpha_{6}\rangle =\frac{1}{\sqrt{2}}\left(|H\rangle |V\rangle |H\rangle -|V\rangle |H\rangle |V\rangle \right)=\frac{1}{2}\left(|++-\rangle -|+-+\rangle +|-++\rangle -|---\rangle \right)\\ |\alpha_{7}\rangle =\frac{1}{\sqrt{2}}\left(|H\rangle |H\rangle |V\rangle +|V\rangle |V\rangle |H\rangle \right)=\frac{1}{2}\left(|+++\rangle -|+--\rangle -|-+-\rangle +|--+\rangle \right)\\ |\alpha_{8}\rangle =\frac{1}{\sqrt{2}}\left(|H\rangle |H\rangle |V\rangle -|V\rangle |V\rangle |H\rangle \right)=\frac{1}{2}\left(|++-\rangle -|+-+\rangle -|-++\rangle +|---\rangle \right) \end{array}$$

Taking $|\alpha_{1}\rangle$ as an example, it will randomly collapse into $|\gamma_{1}\rangle =|H\rangle |H\rangle |H\rangle$ or $|\gamma_{2}\rangle =|V\rangle |V\rangle |V\rangle$ in the Z basis. In the X basis, any user obtains $|+\rangle$ ($|-\rangle$) when the other obtains the same (different) polarization. We use the character that provides the security of the GHZ entangled sources to generate a secure key in the Z basis and error detection in the X basis.

Based on the principle of MDI-QKD [7], our scheme can resist attacks on the measurement devices. In addition to resisting attacks on the measurement device, our scheme can resist a PNS attack using the decoy state technique [22,25,28,29]. Users can employ weak coherent sources with a decoy-state. The GHZ entangled state is equivalent to an ideal single-photon state in each quantum channel. A PNS attack is ineffective for an ideal single-photon source. Therefore, our scheme can resist PNS attacks.

Compared with traditional MDI-QKD, ref. [30] reported the longest communication record that reached 404 km in experiments, while the simulation result shows that our protocol can be utilized with greater than 560 km between each two users. Four detectors are required to build a traditional MDI-QKD system between two users. Therefore, 2n (n − 1) detectors are required to establish a quantum communication system using the MDI-QKD protocol between n users. However, in our protocol, the number of detectors required to establish communications between n users is reduced to only 4n. Moreover, only 2n channels rather than n (n − 1)/2 channels are required if a traditional point-to-point protocol [7] is used between the users and the measurement device. Thus, the cost and complexity of the network are reduced.

We compare our scheme with other multi-user schemes in Table 3. When we employ a single photon source with $\eta_{d}=93\%$, the available distance can reach more than 560 km between each two users. When we employ a weak coherent source with $\eta_{d}=93\%$, the available distance can reach more than 420 km between each two users.

Unlike the MDI-QCC based on a post-selection GHZ entangled state [26] and PM-QCC based on a post-selection GHZ entangled state [19], our protocol uses a GHZ entangled state and the polarization state prepared by users to execute BSM and realize multi-user sharing of a common secret key.

CKA schemes [32] based on the principle of twin-field QKD [33] can realize a high secure key rate and long communication distance through the single photon interference. In our scheme, with a more flexible number of users, we can increase the distance between the GHZ-ESS and the measurement device to enhance the communication distance between each two users. In addition, we can expand our scheme further into a multi-node quantum network, as detailed in Section 4.

Because of the coincidence measurement at the measurement device, increasing the number of users will lead to an obvious decrease in the secure key rate. We propose a system that uses an adaptive technique in Appendix B, while we need to investigate the specific performance in our scheme. At the same time, we will consider using asynchronous time multiplexing technology [34,35], which idea is based on adaptive techniques, to further improve our scheme through enhancing the secure key rate under multi-user scenarios.

## 4. Expansion of Our Protocol

**Figure 5:**
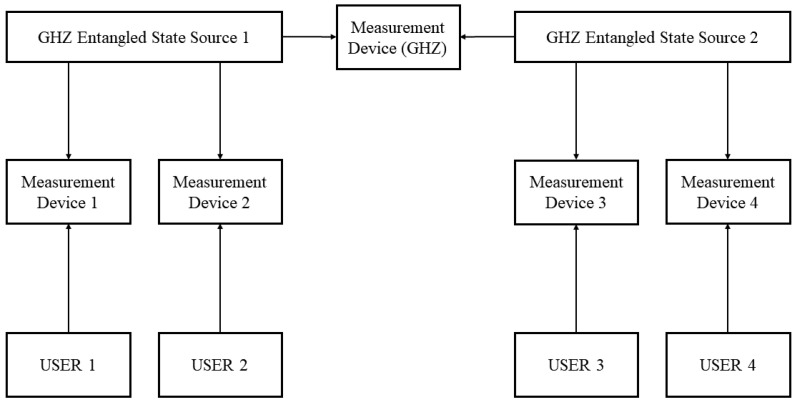
An example of two users per node. The system can help user 1, user 2, user 3, and user 4 to share a common secure key. Unlike our system shown in Figure 1, we build the relationship between two GHZ-ESSs with measurement devices that use BSM like ordinary MDI-QKD devices.

Based on the location-changeable GHZ-ESS, we can expand our scheme further into a multi-node quantum network without quantum memory. An example of two nodes with two users per node is shown in Figure 5, and we can extend the system to n nodes with n users per node.

In the system, we use measurement devices that perform BSM to construct the entangled relationship between two adjacent GHZ entangled sources and extend the communication distance between users in different nodes by increasing the distance between the GHZ-ESS and the measurement device. We use the example shown in Figure 5 to detail the process. In theory, the longest secure communication between user 3 and user 1 can be estimated as follows:

(15) $$L_{U_{1}U_{3}}=L_{U_{1}M_{1}}+L_{GHZ_{1}M_{1}}+L_{GHZ_{1}M_{GHZ}}+L_{GHZ_{2}M_{GHZ}}+L_{GHZ_{2}M_{3}}+L_{U_{3}M_{3}}$$

where $L_{U_{1}M_{1}}$ ($L_{U_{3}M_{3}}$) is the distance between the user and the corresponding measurement device, $L_{GHZ_{1}M_{1}}$ ($L_{GHZ_{2}M_{3}}$) is the distance between the GHZ-ESS and the corresponding measurement device, and $L_{GHZ_{1}M_{GHZ}}$ ($L_{GHZ_{1}M_{GHZ}}$) is the distance between the GHZ-ESS and the measurement device that is between the adjacent GHZ-ESSs.

When we assume that two GHZ-ESSs prepare the same GHZ entangled state and all measurement devices obtain successful BSM events ($|\psi^{+}\rangle$ or $|\psi^{-}\rangle$), the operations of users in the Z basis are as shown in Table 4.

## 5. Conclusions

In this study, we presented a multi-user MDI-QKD scheme based on the GHZ entangled state. We analyzed the security of our scheme and derived the secure key rate when users use an ideal single photon source and a weak coherent source. The MDI-QKD-based scheme is also immune to attacks on the measurement devices, and the communication distance is increased. Furthermore, in contrast to the multi-user quantum network implemented by the original MDI-QKD protocol, the number of detectors required in our scheme is reduced from 2n (n −1 ) to 4n, and the number of quantum channels is reduced from n (n − 1)/2 to 2n. Our scheme realizes an ultra-long QKD available communication distance that can reach more than 280 km between each user and measurement device (i.e., the longest communication distance between any two users can reach more than 560 km) and further extend by changing the location of the GHZ-ESS. In addition, we can expand our scheme further to a multi-node quantum network without quantum memory, which enhances the communication distance between two users.

Although our scheme can be flexibly applied to QKD networks, there are still two issues remaining to be studied in the future. On one hand, the location of the GHZ entangled source can be changed in our scheme, and we can extend the communication distance between two users by increasing the distance between the GHZ entangled source and the measurement device. Therefore, we will study the influence of GHZ entangled state long-distance division. On the other hand, in our estimation of the secure key rate, we assume that the distance between each user and the corresponding measurement device is similar. Therefore, it will be interesting to consider an asymmetric situation.

## Figures and Tables

**Table 1:** Participants and their operations in the Z basis.

GHZ State	BSM 1	BSM 2	BSM 3	Alice	Bob	Charles
$\frac{1}{\sqrt{2}}\left(|H\rangle |H\rangle |H\rangle \pm |V\rangle |V\rangle |V\rangle \right)$	$|\psi^{\pm }\rangle$	$|\psi^{\pm }\rangle$	$|\psi^{\pm }\rangle$	No Flip	No Flip	No Flip
$\frac{1}{\sqrt{2}}\left(|H\rangle |V\rangle |H\rangle \pm |V\rangle |H\rangle |V\rangle \right)$	$|\psi^{\pm }\rangle$	$|\psi^{\pm }\rangle$	$|\psi^{\pm }\rangle$	No Flip	Flip	No Flip
$\frac{1}{\sqrt{2}}\left(|H\rangle |H\rangle |V\rangle \pm |V\rangle |V\rangle |H\rangle \right)$	$|\psi^{\pm }\rangle$	$|\psi^{\pm }\rangle$	$|\psi^{\pm }\rangle$	No Flip	No Flip	Flip
$\frac{1}{\sqrt{2}}\left(|H\rangle |V\rangle |V\rangle \pm |V\rangle |H\rangle |H\rangle \right)$	$|\psi^{\pm }\rangle$	$|\psi^{\pm }\rangle$	$|\psi^{\pm }\rangle$	No Flip	Flip	Flip

**Table 2:** Experimental parameters used in the simulation. **$e_{d}$** is the system intrinsic bit error rate, **$P_{d}$** is the dark count, ***f*** is the error correction inefficiency, and **α** is the optical fiber transmission loss.

$e_{d}$	$P_{d}$	*f*	α
2%	$8\times 10^{-8}$	1.16	0.2

**Table 3:** Comparison of multi-user schemes.

Items	GHZ State MDI-QCC [26]	W State Multi-User MDI-QKD [31]	GHZ State Multi-User MDI-QKD [23]	Our Scheme
Entangled State	GHZ state	W state	GHZ state	GHZ state
Users	≥3	≥4	≥3	≥3
Secure Key Rate	$10^{-16}$ ($\eta_{d}=93\%$, 400 km between two users, weak coherent)	$10^{-16}$ ($\eta_{d}=93\%$, 260 km between two users, single photon)	$10^{-21}$ ($\eta_{d}=40\%$, 400 km between two users, single photon)	$10^{-15}$ ($\eta_{d}=93\%$, 400 km between two users, single photon)
Available Distance	420 km between two users (weak coherent)	260 km between two users (single photon)	520 km between two users (single photon)	560 km between two users (single photon) 420 km between two users (weak coherent)

**Table 4:** The operations of users in different GHZ states in the Z basis. The operation of bit flip according to user 1 and all measurement devices obtain successful BSM events ($|\psi^{+}\rangle$ or $|\psi^{-}\rangle$). We assume that the first particle of the first GHZ is entangled, corresponding to measurement device 1, and the first particle of the second GHZ entangled state corresponds to measurement device 3.

GHZ state	USER 1	USER 2	USER 3	USER 4
$\frac{1}{\sqrt{2}}\left(|H\rangle |H\rangle |H\rangle \pm |V\rangle |V\rangle |V\rangle \right)$	No Flip	No Flip	Flip	Flip
$\frac{1}{\sqrt{2}}\left(|H\rangle |V\rangle |H\rangle \pm |V\rangle |H\rangle |V\rangle \right)$	No Flip	Flip	Flip	No Flip
$\frac{1}{\sqrt{2}}\left(|H\rangle |H\rangle |V\rangle \pm |V\rangle |V\rangle |H\rangle \right)$	No Flip	No Flip	Flip	Flip
$\frac{1}{\sqrt{2}}\left(|H\rangle |V\rangle |V\rangle \pm |V\rangle |H\rangle |H\rangle \right)$	No Flip	Flip	Flip	No Flip

**Figure A1:**
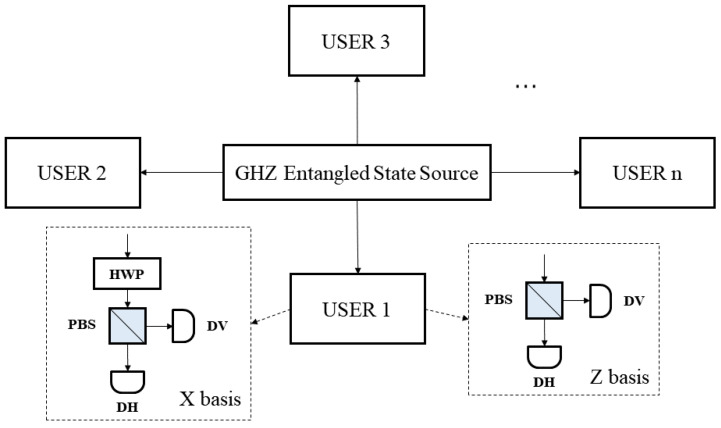
The equivalent topological schematic diagram of the GHZ entangled source distributing a particle of the n-particle GHZ entangled states to each user. **HWP**: half wave plate; **PBS**: polarization beam splitter; **DH**, **DV**: single photon detector.

**Figure A2:**
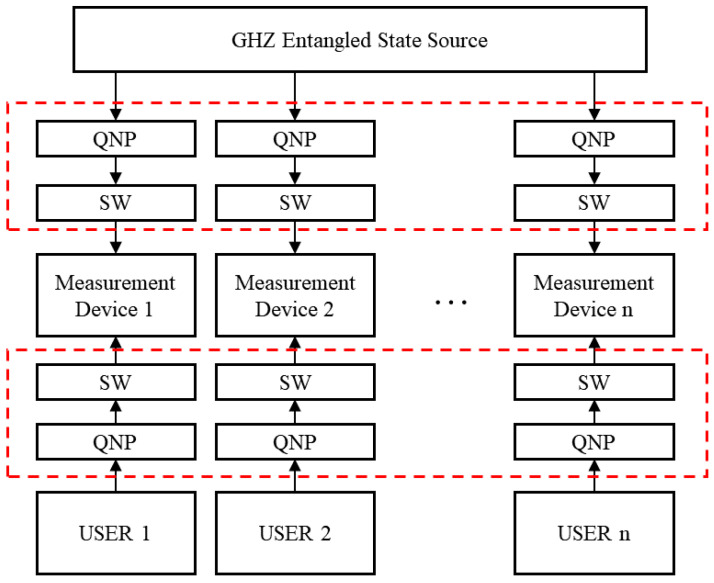
Multi-user MDI-QKD system with adaptive method. **QNP**: quantum non-demolition measurement; **SW**: optical switches. The adaptive technology structures are shown inside the red dashed frames.

## Data Availability

Not applicable.

## Appendix A. Protocol Analysis

In this section, we analyze the generation of the secure key. The key generation in the Z basis in our protocol can be equivalent to the construction shown in Figure A1. The GHZ entangled source distributes a particle of the n-particle GHZ entangled states to each user. Users measure the polarization of the particle and obtain the secure key.

We also considered the case of three users. As shown in Table A1, the GHZ-ESS prepares different GHZ states and sends them to users. Users measure the polarization in the Z basis. They obtain value “0” when the measurement result is $|H\rangle$, and value “1” corresponds to $|V\rangle$.

**Table A1:** Measurement result and value of the secure key. **GHZ entangled state** is the GHZ state sent by the entangled source. **MR1**, **MR2**, and **MR3** are the measurement results of user 1, user 2, and user 3. and **Value1**, **Value2**, and **Value3** are the values of the users’ secure keys.

GHZ Entangled State	MR1	Value1	MR2	Value2	MR3	Value3
$\frac{1}{\sqrt{2}}\left(|H\rangle |H\rangle |H\rangle \pm |V\rangle |V\rangle |V\rangle \right)$	$|H\rangle$	0	$|H\rangle$	0	$|H\rangle$	0
	$|V\rangle$	1	$|V\rangle$	1	$|V\rangle$	1
$\frac{1}{\sqrt{2}}\left(|V\rangle |H\rangle |H\rangle \pm |H\rangle |V\rangle |V\rangle \right)$	$|V\rangle$	1	$|H\rangle$	0	$|H\rangle$	0
	$|H\rangle$	0	$|V\rangle$	1	$|V\rangle$	1
$\frac{1}{\sqrt{2}}\left(|H\rangle |V\rangle |H\rangle \pm |V\rangle |H\rangle |V\rangle \right)$	$|H\rangle$	0	$|V\rangle$	1	$|H\rangle$	0
	$|V\rangle$	1	$|H\rangle$	0	$|V\rangle$	1
$\frac{1}{\sqrt{2}}\left(|H\rangle |H\rangle |V\rangle \pm |V\rangle |V\rangle |H\rangle \right)$	$|H\rangle$	0	$|H\rangle$	0	$|V\rangle$	1
	$|V\rangle$	1	$|V\rangle$	1	$|H\rangle$	0

Compared with the equivalent topological scheme, our protocol can resist attacks on measurement devices and enhance the communication distance between two users. Moreover, if we locate the GHZ entangled source near the measurement device, can we greatly reduce the effect of decoherence of GHZ entangled states. However, we can also increase the distance between the GHZ-ESS and measurement device to increase the communication distance between the two users. Therefore, our protocol can realize multi-user QKD over an ultra-long distance.

## Appendix B. Increasing the Secure Key Rate with an Adaptive Technique

According to the idea in [36], we propose our multi-user MDI-QKD system with an adaptive technique as shown in Figure A2.

The implementation process is as follows. Firstly, all users and GHZ-ESSs send many signal states to the corresponding measurement device; secondly, the QNP performs the quantum non-demolition measurement to detect the arrival of the signal; thirdly, the SW is used to match the arrival signal from the GHZ-ESS and users; fourthly, the measurement device performs BSM between the matched signals and broadcasts the matched result as well as measurement result; lastly, the user keeps the signal data that relate to successful BSM events and other post-processing.

Through the use of an adaptive technique, we can enhance the secure key rate in our multi-user QKD scheme. However, the lack of any particles in the GHZ entangled state will lead to the entire entangled state being unusable, and the GHZ-ESS will need to prepare more signal states than users in principle. We will study the specific performance of using an adaptive technique in future research.
